# Catastrophic health expenditure and health-related quality of life among older adults in Shandong, China: the moderation effect of daily care by adult children

**DOI:** 10.1186/s12939-023-02057-4

**Published:** 2024-03-11

**Authors:** Jiayan Li, Tingting Gao, Dan Zhao, Shujun Chai, Jingjing Luo, Xuehong Wang, Xueqing Wang, Jingjie Sun, Peilong Li, Chengchao Zhou

**Affiliations:** 1 Centre for Health Management and Policy Research, School of Public Health, Cheeloo College of Medicine, Shandong University, 44 Wen-hua-xi Road, Jinan, Shandong 250012 China; 2 Shandong Health Commission Medical Management Service Center, Jinan, 250012 China; 3 Institute of Health and Elderly Care, Shandong University, Jinan, 250012 China; 4 NHC Key Lab of Health Economics and Policy Research, Shandong University), Jinan, 250012 China

**Keywords:** Catastrophic health expenditure, Health-related quality of life, Adult children, Daily life caregiver

## Abstract

**Background:**

Catastrophic health expenditure (CHE) has a considerable impact on older people in later life, but little is known about the relationship between catastrophic health expenditure and health-related quality of life (HRQOL). The aim of this study was to examine the relationship between catastrophic health expenditure and health-related quality of life in older people, and to explore whether the daily care provided by adult children is a moderator in this relationship.

**Methods:**

Data from the sixth National Health Services Survey in Shandong Province, China. The sample consisted of 8599 elderly people (age ≥ 60 years; 51.7% of female). Health-related quality of life was measured by the health utility value of EQ-5D-3 L. Interaction effects were analyzed using Tobit regression models and marginal effects analysis.

**Results:**

The catastrophic health expenditure prevalence was 60.5% among older people in Shandong, China. catastrophic health expenditure was significantly associated with lower health-related quality of life (*β*= − 0.142, *P* < 0.001). We found that adult children providing daily care services to their parents mitigated the effect of catastrophic health expenditure on health-related quality of life among older people (*β* = 0.027, *P* = 0.040).

**Conclusions:**

Our findings suggested that catastrophic health expenditure was associated with health-related quality of life and the caring role of older adult children moderated this relationship. Reducing the damage caused by catastrophic health expenditure helps to improve health-related quality of life in older people. Adult children should increase intergenerational contact, provide timely financial and emotional support to reduce the negative impact of catastrophic health expenditure on health-related quality of life.

## Introduction

Population ageing has become a global issue and the situation in China is serious. A total of 264 million people aged 60 and above live in China in 2020, accounting for 18.7% of the total population, and this number is expected to reach a peak of 487 million in 2053 [1]. As the older population grows and life expectancy increases, improving the health-related quality of life (HRQOL) has become one of the overarching goals of the World Health Organization’s (WHO) ‘Healthy People 2020’ framework and deserves a public health priority [2]. HRQOL is a multidimensional indicator of health that includes physical function, mental health and an individual’s perceived socially relevant role over time [3]. HRQOL has been shown to be associated with multiple adverse health events, for example, as HRQOL decreases, health service utilization increases significantly [4]. Low HRQOL increases health care costs and economic burden [5]. Meanwhile, HRQOL is also an important independent risk factor for mortality in the elderly [6]. Therefore, exploring risk factors for HRQOL deserves more attention, which is essential for developing promising interventions to improve overall well-being of older people in later life.

The factors affecting HRQOL are also multifaceted, including age, chronic illness, average monthly income, and medical expenses [7–9]. Of which, financial hardship is associated with deterioration in a range of health outcomes in later life, which exacerbates HRQOL [10]. The financial hardship caused by high medical costs is often catastrophic. Catastrophic health expenditure (CHE) is defined as health expenditure that exceeds a predetermined percentage of a household’s ability to pay for health care [11]. Previous studies have focused more on the relationship of CHE (or the financial burden of healthcare) with mental health [12–14]. Some studies have shown that high medical expenditure was associated with poor quality of life in patients with type II diabetes [15]. A study from Korea indicated that people who experienced CHE tended to report lower health utility values compared to those without CHE [16]. Existing studies have mostly focused on the impact of chronic disease or health insurance on the relationship between CHE and HRQOL, and there is a lack of research on the role of caregivers of older adults. Although the association between CHE and HRQOL has been confirmed, the underlying mechanism is still unclear.

In China, as formal care services are not yet widely available, most older people have to rely heavily on informal care services provided by spouses, adult children or other family members [17]. Previous research [18] has shown that a network of caregivers, including adult children and friends, moderates the relationship between cancer-related financial difficulties and quality of life. The impact of this connection may vary according to the type of support provided, interpersonal dynamics, and other factors, where differences in the roles of different caregivers have not been clarified. Prior research confirmed that financial aid from their offspring played a vital role in the quality of life for parents [19]. Tang et al. proposed that emotional support from adult children were effective in reducing the risk of CHE in middle-aged and older families [20]. These studies found that the daily care provided by adult children has a positive effect on the health of the elderly, which can reduce the impact of financial difficulties and improve the quality of life of the elderly. However, care provided by other people may lead to different results. Older people may react negatively to the daily care provided by their spouse, and these negative reactions may affect HRQOL [21]. The effect of the care services provided by community or professional nursing staff on HRQOL in older people may not be significant [22]. Few studies have explored whether the caring role of adult children moderates the relationship between CHE and HRQOL in older adults, it remains an undervalued and understudied topic.

The objectives of this study are as follows. First, to examine the relationship between the experience of CHE and HRQOL in older people. Second, to explore whether the daily care provided by adult children is a moderator of the relationship between CHE and HRQOL. This study will provide new perspectives to improve the quality of life of older people.

## Method

### Design and sample

Data for this study were from the sixth Health Services Survey of Shandong Province, China, 2018, which is part of the National Health Services Survey (NHSS). The NHSS is a nationally representative survey conducted by the National Health Commission every five years since 1993, with the aim of fully understand the health and health service needs in the Chinese population [23]. According to the Health Commission of Shandong Province 2020 survey data, the population aged 60 years and above reached 21.22 million, accounting for 20.90% of the total population. A multi-stage whole-group sampling method was used to select study sample. Firstly, 20 counties were randomly selected from 137 counties in Shandong Province. Secondly, five townships were randomly selected from each county, and two sample villages (communities) were randomly selected from each township. Thirdly, 60 or more households were surveyed in each sample village. Totally, 100 townships and 200 villages were selected, comprising 12,938 households and 35,264 individuals.

### Sample selection

**Fig. 1:**
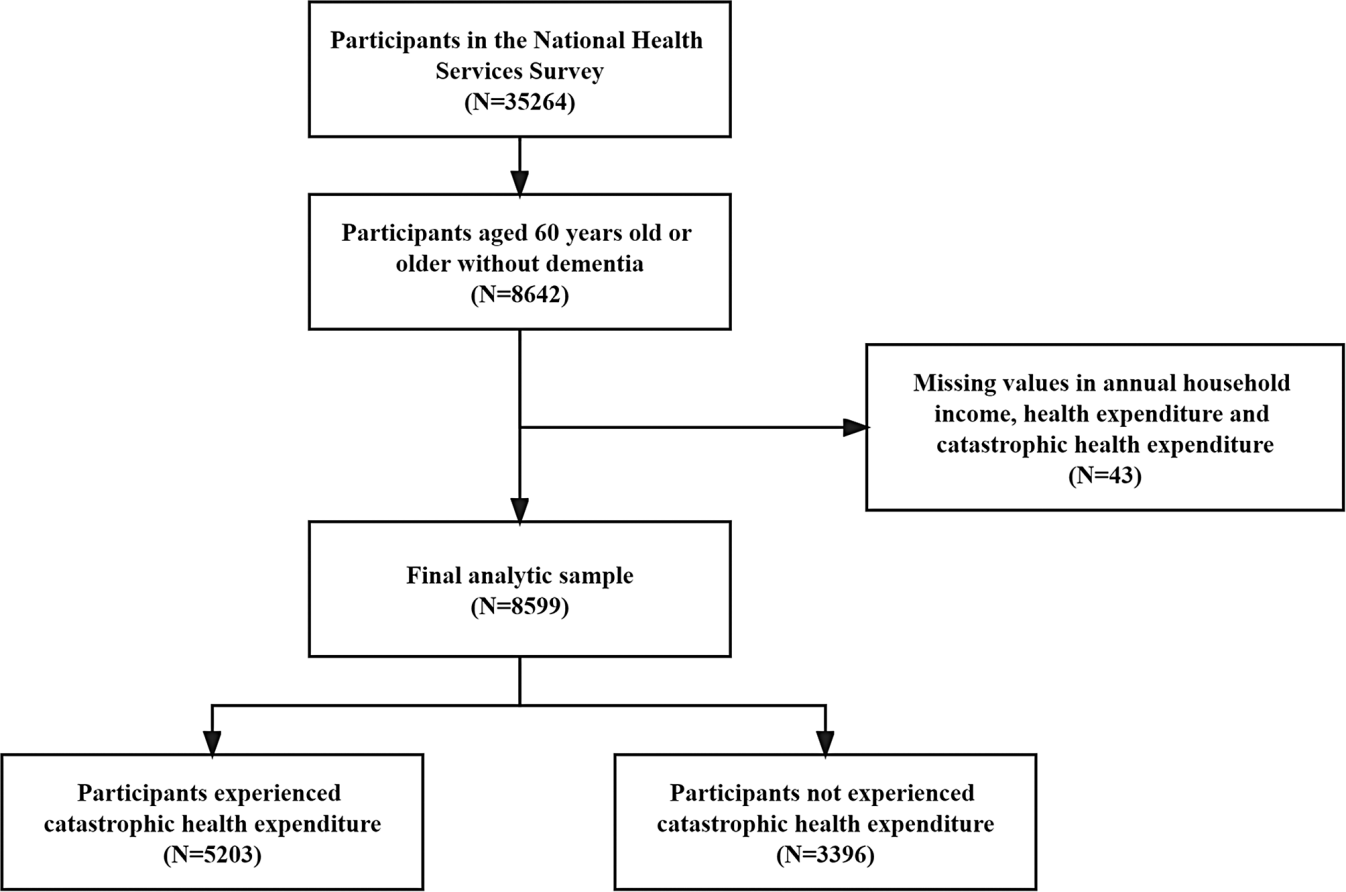
Flowchart of the study sample

The data was collected through face-to-face interviews between September and October 2018. Trained investigators interviewed all household members using a structured questionnaire. As the focus was on the older population, we restricted the age of respondents at 60 or older. We excluded 261 subjects with dementia and 43 participants with missing information on annual household income, household health expenditure and CHE from a total of 8,903 older people interviewed. The final 8,599 older adults were included in the study. Figure 1 shows a flow chart of the study population selection.

### Measurement

#### Health-related quality of life

HRQOL is assessed using the EQ-5D-3 L questionnaire, which consists of five health dimensions (mobility, self-care, daily activities, pain/discomfort, and anxiety/depression). Each of the dimension has three levels of questions, indicating no problem, moderate problem or severe problem. The EQ-5D-3 L utility values were obtained by using a time trade-off model set up for the general Chinese population [24]. It is generated by weighting each dimension of HRQOL, ranging from − 0.149 to 1.0, with higher EQ-5D-3 L utility values representing higher HRQOL [25, 26].

#### Catastrophic health expenditure

CHE was a binary variable: occurring it or not. According to the recommendation by WHO, household health care expenditure equal to or exceeding 40% of a household’s ability to pay is considered catastrophic [27]. Based on this definition, household affordability is defined as total household expenditure less food expenditure [28]. Total household expenditure is the total consumption expenditure of the respondent’s household in the year before the survey, including consumption expenditure on food, tobacco and alcohol, clothing, housing, household goods and other items. Household health care expenditure includes total purchases of medical devices, medicines, medical services and health care appliances, supplies and services. The calculation of CHE is described in detail elsewhere by Wagstaff et al. [29].

#### Caregivers of older adults

The self-report measure was used to assess the caregivers of older people in their daily lives. All respondents were asked “Who would be the main person to help you when you need care?” A total of ten options were provided: “spouse”; “adult children”; “kinsfolk”; “neighbors”; “house maid”; “community workers”; “elderly care facility (caregiver) ”; “medical staff”; “other”; “none”. This study focused on the daily care of older people by their adult children, which was traditionally the main living arrangement for older people in China. Therefore, we divided the caregivers into two categories, adult children and others [30, 31].

#### Covariates

We included socio-demographic, health-related and socio-economic variables as covariates. Socio-demographic variables included gender, age, marital status, region, educational attainment and employment status. Health-related variables included the number of chronic diseases, physical activity, cigarette smoking, alcohol drinking, disability status, body mass index (BMI) and physical examination. Physical activity was divided into two categories based on the following questions: how many times a week you consciously exercised on average in the past 30 days and the average duration of exercise. 3 or more times a week and each time for more than 30 min, coding yes, otherwise no. Disability status was assessed by the Activities of Daily Living Scale [32], including bathing, dressing, toileting, continence control, getting in and out of bed and eating. People who completed all six activities without help were coded no, otherwise yes. Socio-economic variables included basic medical insurance, critical illness insurance and commercial medical insurance.

### Data analysis

All statistical analyses were performed using Stata 14.0. Confidence intervals for reporting were calculated at the 95% level and *p*-values less than 0.05 were considered statistically significant. Firstly, we used descriptive analysis to describe the overall characteristics of the sample. Secondly, one-way ANOVA was used to compare EQ-5D utility values between the two groups (incurred and not incurred CHE) of older adults. Since the EQ-5D scale had a strong ceiling effect, that is, most respondents reported “no problem” in each dimension, the range of the calculated dependent variable was limited and had the characteristics of being intercepted, we used Tobit regression model to examine the relationship between the occurrence of CHE and HRQOL. Model 1 of the regression analysis included only the CHE unadjusted, and Model 2 included the adjusted covariates. Finally, we explored whether caring role of older people’s adult children was a potential moderator of this relationship. We included an interaction term (catastrophic health expenditure × caregivers) in model 3 to test the moderating effect of the daily care provided by adult children in the association between CHE and HRQOL. The margins plot was used to illustrate the predictions of CHE and caregiver category on HRQOL.

## Results

### Characteristics of participants

Table 1 presents the basic characteristics of the participants. Of the 8,599 older people aged 60 and over, 60.5% had CHE and 45.8% had adult children as their primary caregivers in their daily lives. Respondents had an average age of 68.5 years and were mostly female (51.7%), married (84.0%), with secondary school education or less (63.3%) and suffering from chronic disease (55.1%).

**Table 1:** Descriptive analysis of catastrophic health expenditure among older adults in Shandong, China, 2018 (N = 8599)

Characteristics	N (%)	Catastrophic health expenditure		*p-*value
		No (%)	Yes (%)	
Gender				0.037
Male	4,152 (48.3)	1,687 (40.6)	2,465 (59.4)	
Female	4,447 (51.7)	1,709 (38.4)	2,738 (61.6)	
Age, mean (SD)	68.53 ± 6.75	68.06 ± 6.72	69.62 ± 6.69	< 0.001
Marital status				0.001
Married	7,227 (84.0)	2,797 (38.7)	4,430 (61.3)	
Unmarried or others ^a^	1,372 (16.0)	599 (43.7)	773 (56.3)	
Region				< 0.001
Urban areas	4,187 (48.7)	1,833 (43.8)	2,354(56.2)	
Rural areas	4,412(51.3)	1,563 (35.4)	2,849 (64.6)	
Education				< 0.001
Illiterate	2,647 (30.8)	872 (32.9)	1,775 (67.1)	
Primary school	2,795 (32.5)	1,127 (40.3)	1,668 (59.7)	
Middle school or above	3,157 (36.7)	1,397 (44.3)	1,760 (55.7)	
Employment status				0.119
Unemployed	6,062 (70.5)	2,306 (38.0)	3,756 (62.0)	
Employed	2,537 (29.5)	1,090 (43.0)	1,447 (57.0)	
Number of chronic diseases				< 0.001
0	3,856 (44.8)	1,880 (48.8)	1,976 (51.2)	
1	2,996 (34.8)	1,033 (34.5)	1,963 (65.5)	
≥ 2	1,747 (20.3)	483 (27.6)	1,264 (72.4)	
Exercise				< 0.001
No	4,537 (52.8)	1,684 (37.1)	2,853 (62.9)	
Yes	4,062 (47.2)	1,712 (42.1)	2,350 (57.9)	
Smoking status ^b^				< 0.001
No	6,907 (80.3)	2,663 (38.6)	4,244 (61.4)	
Yes	1,692 (19.7)	733 (43.3)	959 (56.7)	
Alcohol drinking ^c^				< 0.001
No	6,548 (76.1)	2,441 (37.3)	4,107 (62.7)	
Yes	2,051 (23.9)	955 (46.6)	1,096 (53.4)	
Physical disability				< 0.001
No	7,330 (85.2)	3,056 (41.7)	4,274 (58.3)	
Yes	1,212 (14.1)	313 (25.8)	899 (74.2)	
Body mass index				0.005
< 18.5	491 (5.7)	170 (34.6)	321 (65.4)	
18.5 ~ 24.0	3,839 (44.6)	1,480 (38.6)	2,359 (61.4)	
24.0 ~ 28.0	3,124 (36.3)	1,303 (41.7)	1,821 (58.3)	
≥ 28.0	1,145 (13.3)	443 (38.7)	702 (61.3)	
Physical examination ^d^				< 0.001
No	2,395 (27.9)	1,062 (44.3)	1,333 (55.7)	
Yes	6,204 (72.1)	2,334 (37.6)	3,870 (62.4)	
Basic medical insurance				0.954
No	93 (1.1)	37 (39.8)	56 (60.2)	
Yes	8,506 (98.9)	3,359 (39.5)	5,147 (60.5)	
Critical illness insurance				< 0.001
No	2,852 (33.2)	1,237 (43.4)	1,615 (56.6)	
Yes	5,747 (66.8)	2,159 (37.6)	3,588 (62.4)	
Commercial medical insurance				0.191
No	8,139 (94.7)	3,201 (39.3)	4,938 (60.7)	
Yes	460 (5.3)	195 (42.4)	265 (57.6)	
Caregivers				0.83
Spouse or others	4,662 (54.2)	1,846 (39.6)	2,816 (60.4)	
Older people’s adult children	3,937 (45.8)	1,550 (39.4)	2,387 (60.6)	

Note:

^a^ Others include those who are divorced (34, 0.40%), widowed (1222, 14.31%) and other situations (15, 0.18%)

^b^ Current smokers were coded as yes, never smokers and quit smokers as no

^c^ Current drinkers were coded as yes, never drinkers and abstinence as no

^d^ Those who had received a health examination in the last 12 months were coded yes, otherwise no

### EQ-5D-3 L values by different CHE status

As shown in Table 2, the mean EQ-5D utility value was 0.887 (± 0.177). Independent sample t-tests showed that EQ-5D-3 L utility values differed significantly between CHE states (*p* < 0.05). Mean EQ-5D-3 L utility values were higher in patients who did not experience CHE than in those who did. The CHE group reported more problems across all dimensions (mobility, self-care, usual activity, pain/discomfort, and anxiety/depression). When stratified by different caregiver roles, EQ-5D-3 L utility values differed across CHE states for older people’s adult children and other caregivers.

**Table 2:** Observed utility values of EQ-5D-3 L of older adults by catastrophic health expenditure and caregivers in Shandong, China, 2018

Characteristics	Mean ± SD ^a^	EQ-5D-3 L (%) ^b^				
		Mo	SC	UA	PD	AD
Total	0.887 ± 0.177	20.4	8.9	13.8	34.5	10.3
CHE ^c^						
No CHE	0.921 ± 0.150	14.3	5.7	9.0	25.4	6.4
Occur CHE	0.865 ± 0.189^***^	24.5	11.0	16.9	40.5	12.9
Caregivers						
Older people’s adult children						
No CHE	0.909 ± 0.159	16.9	6.5	11.0	28.0	7.2
Occur CHE	0.863 ± 0.183^***^	25.4	11.1	17.0	41.6	13.1
Spouse or others						
No CHE	0.931 ± 0.141	12.0	5.1	7.4	23.2	5.6
Occur CHE	0.867 ± 0.194^***^	23.7	10.8	16.8	39.5	12.7

Note: HRQOL Health-related quality of life, MO Mobility, SC Self-care, UA Usual activity, PD Pain/discomfort, AD Anxiety/depression

*p*-values with statistical significance: ^*^p < 0.05, ^**^p < 0.01, ^***^p < 0.001

^a^ Observed EQ-5D-3 L utility values; SD: standard deviation

^b^ Observed frequency (%) of “have problems” in EQ-5D-3 L dimensions

^c^ Independent-samples t-test was used to compare the EQ-5D utility values between different groups

### Association between CHE and HRQOL

As shown in the Table 3, Model 1 showed that older adults who incurred CHE reported significantly lower HRQOL compared to those who did not (*β*= − 0.142, *p* < 0.001). When control variables were added, Model 2 showed that CHE remained significantly associated with HRQOL (*β*= − 0.057, *p* < 0.001), while there was no significant association between caregivers of older people’s daily living and quality of life. In Model 3, a significant interaction term (*β* = 0.027, *p* = 0.040) suggested that adult children providing daily care services to their parents would mitigate the negative effect of CHE on HRQOL in older people.

**Table 3:** Tobit regression models for association between catastrophic health expenditure and HRQOL and its caregivers difference

Characteristics	Model 1		Model 2		Model 3	
	*β* (95%CI)	*p*-value	*β*(95%CI)	*p*-value	*β*(95%CI)	*p*-value
Main terms						
Catastrophic health expenditure						
No	Ref		Ref		Ref	
Yes	-0.142(-0.160, -0.125)	< 0.001	-0.057 (-0.070, -0.043)	< 0.001	-0.067 (-0.088, -0.052)	< 0.001
Caregivers						
Spouse or others			Ref		Ref	
Older people’s adult children			0.010 (-0.004, 0.024)	0.176	-0.008 (-0.030, 0.014)	0.458
Interaction term Catastrophic health expenditure × caregivers						
Catastrophic health expenditure × Spouse or others					Ref	
Catastrophic health expenditure × older people’s adult children					0.027 (0.001, 0.053)	0.040
Age			-0.003 (-0.004, -0.002)	< 0.001	-0.003 (-0.004, -0.002)	< 0.001
Gender						
Male			Ref		Ref	
Female			0.004 (-0.012, 0.020)	0.633	0.004 (-0.013, 0.020)	0.670
Marital status						
Married			Ref		Ref	
Unmarried or others ^a^			-0.024 (-0.043, -0.006)	0.010	-0.024 (-0.042, -0.005)	0.013
Region						
Urban areas			Ref		Ref	
Rural areas			-0.027 (-0.040, -0.014)	< 0.001	-0.027 (-0.040, -0.014)	< 0.001
Education						
Illiterate			Ref		Ref	
Primary school			0.022 (0.006, 0.038)	0.006	0.022 (0.007, 0.038)	0.006
Middle school or above			0.074 (0.056, 0.091)	< 0.001	0.073 (0.055, 0.091)	< 0.001
Employment status						
Unemployed			Ref		Ref	
Employed			0.039 (0.023, 0.054)	< 0.001	0.039 (0.024, 0.054)	< 0.001
Number of chronic diseases						
0			Ref		Ref	
1			-0.078 (-0.093, -0.064)	< 0.001	-0.078 (-0.093, -0.064)	< 0.001
≥ 2			-0.141 (-0.159, -0.124)	< 0.001	-0.141 (-0.158, -0.124)	< 0.001
Exercise						
No			Ref		Ref	
Yes			0.079 (0.065, 0.092)	< 0.001	0.079 (0.065, 0.092)	< 0.001
Smoking status						
No			Ref		Ref	
Yes			-0.008 (-0.027, 0.010)	0.371	-0.009 (-0.027, 0.010)	0.363
Alcohol drinking						
No			Ref		Ref	
Yes			0.047 (0.029, 0.065)	< 0.001	0.046 (0.029, 0.064)	< 0.001
Physical disability						
No			Ref		Ref	
Yes			-0.354 (-0.371, -0.336)	< 0.001	-0.353 (-0.370, -0.336)	< 0.001
Body mass index						
<18.5			Ref		Ref	
18.5 ~ 24.0			0.027 (-0.001, 0.054)	0.043	0.027 (-0.001, 0.054)	0.046
24.0 ~ 28.0			0.041 (0.014, 0.068)	0.003	0.040 (-0.013, 0.068)	0.004
≥ 28.0			0.018 (-0.013, 0.048)	0.258	0.017 (-0.013, 0.047)	0.274
Physical examination						
No			Ref		Ref	
Yes			0.038 (0.023, 0.052)	< 0.001	0.038 (0.023, 0.052)	< 0.001
Basic medical insurance						
No			Ref		Ref	
Yes			0.029 (-0.030, 0.087)	0.337	0.028 (-0.030, 0.087)	0.352
Critical illness insurance						
No			Ref		Ref	
Yes			-0.012 (-0.026, 0.002)	0.086	-0.012 (-0.026, 0.002)	0.088
Commercial medical insurance						
No			Ref		Ref	
Yes			0.005 (-0.024, 0.033)	0.753	0.004 (-0.024, 0.033)	0.769

Note: ^a^ others include those who are divorced (34, 0.40%), widowed (1222, 14.31%) and other situations (15, 0.18%)

## Discussion

This study explored the association between CHE and HRQOL, and the role of daily care provided by adult children in moderating this relationship among older adults. The results of the study showed that the experience of CHE was associated with poorer HRQOL. Furthermore, the relationship between CHE and HRQOL is moderated when the primary caregiver of older people in their daily lives was their adult children. The daily care from adult children reduced the possibility for adverse health effects from CHE.

Our study showed that the incidence of CHE among older people in Shandong province was 60.5%, which was higher than that in previous studies in China. For example, a study using data from China’s Fourth National Health Services Survey (2008) found that the incidence of CHE was 13.0% [33]. It was also higher than the incidence of CHE in a cross-sectional survey conducted in Shandong in 2012 with the prevalence of 44.9% [34]. This may be due to the accelerated aging process and heavy use of health care services by more elderly people, who have lower incomes, thus resulting in an increased incidence of CHE. Another reason might be due to the conservative regional culture in Shandong area, especially in the elderly group. Faced with external investigators, the elderly were reluctant to disclose their real income and expenditure for reasons of personal privacy protection. They tended to underreport their income and overreport their expenditure, which would result in a high calculated CHE prevalence.

Our findings indicated that experiencing CHE is negatively associated with HRQOL in older adult. Respondents who experienced CHE scored significantly lower on each EQ-5D domain and had lower health utility scores than individuals who did not experience CHE. Similar to the previous study, research on the general population showed that people with CHE tended to have lower health utility values than those without CHE, and this association was more pronounced among people with chronic conditions [16]. Some scholars had found that the presence of CHE was associated with poorer physical and mental health among both rural and urban older people in China [14]. Older people who have experienced CHE bear greater subjective and objective financial burdens, and have increased risk of poverty, making them spend less on other aspects of their lives, or reduced the number of medical visits for fear of having to pay high medical costs again, which further lowered health utility values of older people [35].

Our study also showed that the daily care from adult children moderated the association between the CHE and HRQOL. The influence of CHE on HRQOL was larger among older adults who were cared for by their adult children than those who were cared for by non-adult children. Specifically, caregivers appeared to buffer the relationship between CHE and HRQOL. Several possible explanations for this finding are as follows. First, increasing intergenerational contact with adult children not only promotes intra-family relationships, but also significantly improves HRQOL of older people [36]. Second, adult children can provide financial and emotional support to their ageing parents. Transfer payment from adult children significantly reduce poverty among older people [37]. Evidence from rural China suggested that financial support provided by adult children also improves the quality of life of the elderly [38]. Emotional support from adult children can enhance the well-being of older people, improve their life satisfaction [39] and therefore improve HRQOL levels [40]. Third, adult children can share a lot of physical labor for their parents. They are younger and stronger and have more advantages in daily activities, which can help the elderly with heavy work, thus reducing the physical burden of older people [41]. Other carers, such as spouses or relatives, may not be able to provide the desired level of care due to inadequate knowledge of disease management, excessive caregiving burden or disagreements with the older person [42]. In conclusion, older people’s adult children are able to assist them to a large extent in their daily lives, both materially and emotionally. When CHE occur, adult children can compensate their parents financially to ease the financial burden and help them get through a difficult time in life as quickly as possible [37]. Spiritually, adult children can provide emotional support to their parents [43], reduce the psychological distress caused by CHE and improve the HRQOL in the elderly.

Table 1 showed that the incidence of CHE was higher among older adults who have purchased critical illness insurance (62.4%) compared with those who have not (37.6%). This may be because the starting payment line of critical illness insurance is relatively high, and the elderly cannot reach the reimbursement amount for a single medical treatment [44]. Moreover, there is still inequality in medical insurance reimbursement level, services obtained or quality of care, so that the rights and interests of the elderly who have bought insurance have not been effectively protected [45]. Secondly, due to the limited economic conditions or insufficient medical level in the area, the uninsured families actively or passively give up the medical treatment, and there are cases where they should have been hospitalized but were not, their medical needs are not met, so that the risk of CHE in this group of people is underestimated [46].

Based on the findings of the study, we recommend that it is necessary for policy makers to develop intervention mechanisms to protect vulnerable groups from financial risks and thus reduce the incidence of CHE. Firstly, future health policy reforms should take greater account of the affordability of health services and reduce the price of treatment for major diseases and chronic conditions. Secondly, the proportion and scope of health insurance reimbursement should be increased to reduce out-of-pocket costs. For the common chronic diseases and the corresponding treatment drugs in the elderly population, the government should consider setting up reasonable reimbursement rules. Thirdly, as the primary caregivers of the elderly, adult children should pay timely attention to their parents’ physical and psychological status, increase emotional communication, and improve the living conditions, so as to improve the HRQOL of the elderly.

Several limitations of this study also need to be acknowledged. First, due to the possible reciprocal causal relationship between CHE and HRQOL, the cross-sectional data used in this study could not be investigated for rigorous causal studies. We therefore hope to test this relationship using better data and methodology in the future. Second, since self-reported health care expenditure and household income may lead to recall bias, especially in older adults, they are more inclined to overestimate their expenditure and report income to less. Third, this study was applied to older people in Shandong Province, China, and the applicability of the findings to other populations will need to be tested in future studies. Finally, due to the limitations of the sample data used in this study, we did not conduct a classification study of older adults without any caregivers, and in the future, we may screen these special populations on a national level.

## Conclusions

In summary, the prevalence of CHE in older Chinese families is high. Our findings revealed that CHE was negatively associated with HRQOL and the daily care provided by adult children moderated this relationship. Recognizing the relationship between CHE and HRQOL provides policymakers with new insights into poverty prevention. Vulnerable older people should be identified as a target population for priority protection in health policies. In addition, it is important for primary caregivers, especially for adult children, to pay more attention to their parents’ mental health and living environment in order to reduce the incidence of CHE and improve the quality of life of older people.

## Data Availability

The datasets used in the current study are not publicly available due to the confidential policy but are available from the corresponding author on reasonable request.

## Abbreviations

CHE: Catastrophic health expenditure
HRQOL: Health-related quality of life
EQ-5D: EuroQol Five Dimensions Questionnaire
